# Economic valuation from direct use of mangrove forest restoration in Balikpapan Bay, East Kalimantan, Indonesia

**DOI:** 10.12688/f1000research.17012.2

**Published:** 2019-03-29

**Authors:** Abubakar M. Lahjie, Bagus Nouval, Annisa Abubakar Lahjie, Yosep Ruslim, Rochadi Kristiningrum

**Affiliations:** 1 Faculty of Forestry, Mulawarman University, Samarinda, East Kalimantan, 75117, Indonesia; 2 Faculty of Economics and Business, Mulawarman University, Samarinda, East Kalimantan, 75117, Indonesia

**Keywords:** economic valuation, ecosystem services, direct use, mangrove restoration

## Abstract

**Background:** The mangrove forests in Balikpapan Bay, Indonesia, have been used as a source of livelihood for local community more than 150 years. Since the natural products of the mangrove forest, such as wood and seafood, are not able to meet the economic needs of the local community, some areas have been converted into brackish water ponds with traditional aquaculture systems. The growth of brackish water ponds over the last five decades has been identified as the main cause of ecosystem destruction. However, the mangrove ecosystem has been restored naturally through tidal action and seeds falling from mangrove trees.

**Methods:** This study focused on the mangrove tree species *Rhizophora apiculata, *with ages ranging from 3 to 40 years. Initially, the study site (area, 1 ha) was plotted. The study sample size included 30% of the local population, chosen by systematic random sampling. The data collection was undertaken as follows: 1) measurement of the diameter and height of mangrove trees; 2) observation of local fish auctions; and 3) interviewing of fishers and local communities regarding the direct use of the natural products of the mangrove ecosystem.

**Results:** It is suggested that the total income from wood production is IDR 742,425,000 year ^-1^ or US $0.933 person ^-1^ day ^-1^. Furthermore, the total income from fishing is IDR 1,080,353,280 year ^-1^ or US $1.43 person ^-1^ day ^-1^. Pre-thinning income level for wood harvesting is still low. The income difference between wood production and fishing resulted in the rate of overfishing reaching 45.5%. The highest observed wood production was reached at the age of 25 years, and the highest value of mean annual increment (MAI) is 5.39 m ^3 ^ha ^-1 ^at the age of 40 years.

**Conclusions:** This study showed that tree thinning, ranging from 90 to 350 trees ha ^-1^, can increase the value of MAI by around 24.5%.

## Introduction

Mangrove forest are one of the most productive ecosystems worldwide and are located in the brackish water zone of sub-tropical and tropical coastal regions. The ecosystem benefits provided by mangrove forests are not only limited to the provision of habitats for numerous kinds of seafood (including fishes, crustaceans, and molluscs), but also assist with nutrient recycling and soil conservation through sediment trapping ^1^. Furthermore, the economic value of mangrove ecosystems, in terms of wood and seafood production, has provided a major source of income for local communities in coastal areas ^2^. In tropical regions, some mangrove forests have largely disappeared ^3– 6^. The main factors that have caused the destruction of mangrove forests include urban development, development of brackish water ponds, freshwater flows diversion, over-cutting of trees for wood, and development by the local community such as brackish water ponds ^7– 9^. The destruction of mangrove forests has been the major cause of ecosystem loss in developing countries, and it is predicted that mangrove forests will disappear over the next 100 years in sub-tropical and tropical regions ^10^.

There has been increasing awareness among government and local communities of the important role of natural ecosystems in protecting against floods, the reduction of coastal erosion and riverbanks, and in water quality management ^7, 11, 12^. As a consequence, mangrove restoration programs have been proposed in coastal areas where local ecological knowledge has been adopted ^13^. However, these restoration programs have not always been successful ^14, 15^. The failure of restoration programs has caused economic losses of millions of dollars. For example, mangrove restoration in the Philippines has only achieved a 10–20% long-term survival rate for reintroduced species due to inappropriate habitat selection ^16, 17^.

### Defining ecosystem services (ES) and economic valuation (EV)

ES were first defined as the natural functions, consisting of the combination of soil, animals, plants, water, and air, that provide various benefits to society and thereby enhance quality of life for people ^18, 19^. This paper, in line with the Millennium Ecosystem Assessment ^20^, defines ES as goods and services provided by ecosystems and their contributions to the sustenance of human well-being. The benefits of ES for agricultural production (food crops, seafood, medicine, and building materials), maintaining biodiversity (soil production, waste assimilation, and sources of clean water), public policy (microclimate and disease prevention), and intangible aesthetic and cultural benefits (education and recreational projects) are widely acknowledged in the literature ^21– 27^. Furthermore, the Millennium Ecosystem Assessment Board ^28^ categories ES into four groups of services: 1) provisioning (food, freshwater, fuel, wood, and fibre); 2) regulating (disease prevention, water purification, and climate and flood regulation); 3) cultural (aesthetic, spiritual, education, and recreational); and 4) supporting (nutrient cycling, soil formation, and primary production). Although stakeholder groups, such as economists and local communities, depend significantly on the existence of ES for their wellbeing, they often neglect the important of ES. Thus, the value of ES is difficult to estimate ^29^.

Since assessing ecosystem services provided ecological, sociocultural and economic human benefits, and monetary are essential, this research focused on examining the direct use of the natural products of the mangrove ecosystem in order to meet economic needs of the local community. Does definition of ES in this research is limited on the economic services of mangrove ecosystem.

The definition of economic valuation (EV) is a quantitative assessment that is able to present the different range of values based on the specific method used, but it may allow fishermen to make decisions according to utilized goods and services provided by mangrove ecosystems ^23^.

The purposes of this study are to: 1) analyses the production of wood of the *Rhizophora apiculata*; 2) identify the age of trees reached the highest increments of wood of the *Rhizophora apiculata*; 3) measure the highest value of mean annual increments (MAI) of the *Rhizophora apiculata*; and 4) analyse the economic valuation of direct used from the natural productions of mangrove forest in Balikpapan Bay.

## Methods

### Study area

**Figure 1.**
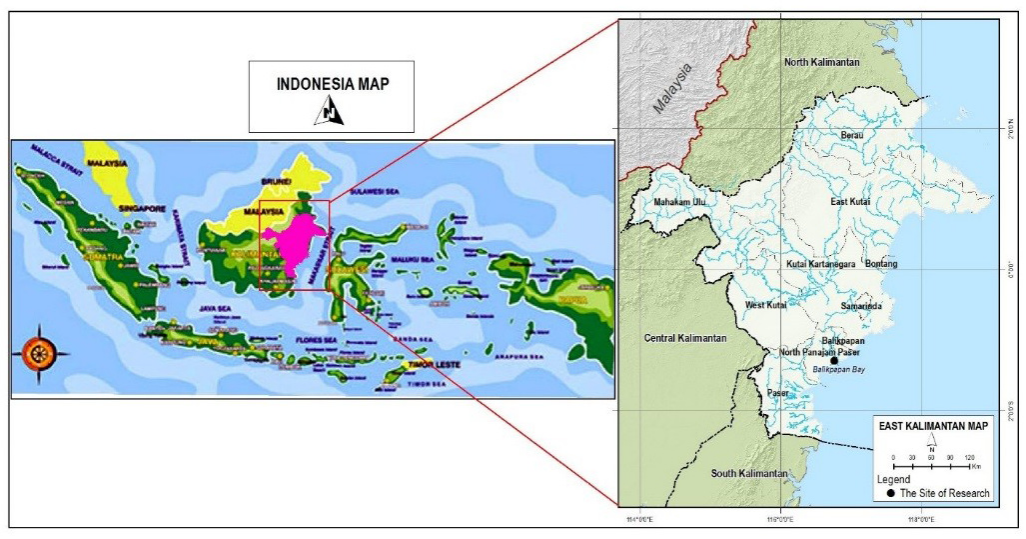
Study area (•) in Balikpapan Bay, East Kalimantan, Indonesia. This figure has been reproduced with kind permission from Muliadi and co-authors ^31^; Lahjie and co-authors ^32^).

Balikpapan Bay is a strategic port in the province of East Kalimantan ( Figure 1). The bay area has 16,000 ha of water, and 156.836 ha of land. Balikpapan Bay has rapidly gained prominence domestically as one of the leading ports of Indonesia. Balikpapan City is now the primary trade and industry centre for mining, fishing, plantation, and forestry in East Kalimantan. With 3.2% population growth per year from 2010 to 2015, around 720,000 people live in Balikpapan. An estimated 108,200 people live in 54 sub-watersheds (more 22 villages) that drain into the bay. As consequence, development in Balikpapan Bay has caused significant ecosystem destruction—approximately 47.6% of the mangrove ecosystem has been lost, and mangrove forests have decreased by around 12.5% in last 15 years. Specifically, mangrove forests decreased from 19,428 ha in 2002 to 17,000 ha in 2017. Furthermore, there has been large-scale habitat destruction that has impacted some species on the endangered species lists, including proboscis monkey *(Nasalis larvatus*), dolphin ( *Orcaella brevirostris*), saltwater crocodile ( *Crocodylus porosus*), and dugong ( *Dugong dugon*).

### Thinning

Four systematically selected 50 m x 50 m plots are established in the study sites from which the increment of wood of the *Rhizophora apiculata* were examined. In particular, in the study sites, each two sampling plots located in different forest monitoring sites (300 m apart) are identified to measure the tree height and diameter for prior and after tree thinning treatment. The sample of this study are selected from 30% of tree population. The method of low thinning was adopted to remove trees which are below two cm from the average of tree diameter.

### Nominal rate of return (NRR)

The principal stated that the rate of return earned on investment should be expressed in the nominal price of Indonesian currency (IDR) (including inflation). Thus, the rate of return is widely known as the nominal rate of return or the nominal interest rate. The principal was proposed by Klemperer ^30^ as the following equation:

$$i=\sqrt[{n}]{\frac{I_{n}}{V_{0}}}-1$$

Where *i* is the inflated or nominal interest rate, *I _n_* is the inflated or current (IDR) value in year *n, n* is the number of years in investment period, V _0_ is the initial value at the start of an investment period.

### Data collection

This study used both direct and indirect approaches to measure the incremental growth of mangrove tree from 2001 to 2018, with trees ranging in age between 3 to 20 years.

For the direct approach methods, this study carried out the following. 1) Measured the diameter and height of mangrove trees to examined biophysical condition of mangrove trees. 2) Observed local fish auctions. 3) Interviewed fishermen and local community members in person at their home in the village and the coastal zone when they catch fishes. Those included must have lived with their family in the community of Kariangau village for more than three decades and made a living as the fisherman for 15 years. This study eliminated fishermen who are categoried as new members of community in Kariangau village, who typically have lived in the village for less than 5 years and work as factory workers in Balikpapan bay, meaning catching fish is not their main income source (under 20% of total income). The topic of interview focuses on the direct use of the natural products of the mangrove ecosystems (e.g., wood and seafood). 4) Undertaken a review of the literature relating to the description of mangrove ecosystem, the restoration of mangroves, and the natural products of mangrove ecosystems in Balikpapan Bay.

Three kinds of observations were made at the fish auction: 1) identify fish species offered; 2) identify the total of sales and the price per kilogram; 3) examine the mechanism of fish auction. The period of observation for sea food production, from fish catch to fish offered in the auction, has been done from April to November 2017.

The data is collected through interview and questionnaire with fisherman in Balikpapan bay, which adopted the technique of accidental and snowball sampling. A copy of the questionnaire is provided on OSF. The period when interviews were conducted was from April to November 2017. The population of fisherman located in the coastal zone of Kariangau village are 40 fishermen. The selected sample is 30 of 40 fishermen population (75%), since they are active fishermen during the interview period. For the indirect approach, government documents regarding the strategic plan in the management of coastal zone in Balikpapan bay has been used as references to present the condition of mangrove forests in Balikpapan bay.

The area utilised for fisheries in mangrove forest covers 300 ha and is located on the border area between Kariangau Village and Batu Ampar Village. The area utilized for fisheries is located at 01° 12′ 50.5″ S, 116° 49′ 26.8″ E. In this study, an exchange rate of US$ 1 = IDR 13,300 has been used, with data supplied by the Indonesian central bank, Bank Indonesia (2018, February).

This study was approved by the general research of Mulawarman University ethics board (approval number 208-41/KL/2017), and each participant gave their written informed consent.

### Data analysis

This study used Microsoft Excel to perform calculations and generate graphs. As Van Gardingen ^33^ argued, there are two proxies that can be adopted to measure the wood production of *Rhizophora apiculata*. The initial proxy for the wood production of *Rhizophora apiculata*, mean annual increment (MAI), is formulated using the total standing volume divided by tree age. The second proxy, period annual increment (PAI), is the absolute difference between total standing volume at age t and age t _-1_, scaled by the time interval between each measurement age. Both MAI and PAI have been used as proxies for wood production by Lahjie ^32^ and Winarni ^34^. The MAI formula is represented as:

$$MAI=\frac{V_{t}}{t}$$

Where MAI is the mean annual increment, Vt is the total standing volume at age t, and t is the tree age.

The PAI formula is represented as:

PAI=Vt−Vt−1t

Where PAI is the period annual increment, Vt is the total standing volume at age t, Vt- _1_ is the total standing volume at age t-1, and t is the time interval between each measurement age.

In examining a single statistical series of R (range) with involved N (sample), the optimal class interval (C) could be approximated using the formula proposed by Sturges ^35^. The formula represents the class interval for measures of the means, dispersion, coefficient of variation, and skewness of the frequency distribution. This can provide the proper distribution into classes for entire numbers, which are powers of two, by a series of binomial coefficients. The Sturges formula is:

$$C=\frac{R}{1+3.332\log N}$$

To analyse the net income of fishers, this study collects data from the population of 40 households (164 people) whose livelihood depends on the natural productivity of the mangrove forests in Balikpapan Bay. Using accidental and snowball sampling method, 30 household (123 people) were further selected for inclusion in the dataset (see Table 1, below).

**Table 1.** The net household income of fishers in Balikpapan bay.

Net household income class	Median income, million IDR	Number of households	People per household	Total no. of people	Annual total net income, million IDR	Daily total net income, US$
13–16	14.5	3	3	9	43.5	0.996
17–20	18.5	4	3	12	74	1.27
21–24	22.5	5	4	20	112.5	1.16
25–28	26.5	8	5	40	212	1.092
29–32	30.5	6	5	30	183	1.26
33–36	34.5	4	3	12	138	2.37
Average		30	23	123	763	1.43

Net income class, million IDR; median income, million IDR; total net income (million) IDR; total net income US$ person ^-1^ day ^-1^; IDR, Indonesian rupiah. 1 US$ = IDR 13,300.

## Result and discussion

All raw data generated in this study are available on OSF ^36^.

### Income of fishermen

Table 1 shows the result of the net income of the fishers based on six class of net income in Balikpapan bay. Table 1 shows that the fishers in Balikpapan Bay have varied net household incomes, which can be divided into six classes. The variability in net household income was influenced by fishing boat capacity, quality of equipment, and number of available working days per year for fishing. The majority of fishers (40 people) had net annual incomes ranging from IDR 25 million to IDR 28 million, with a total net annual income of IDR 212 million. The median net annual income was IDR 26.5 million. The net income per person per day was IDR 18,048 (or US$1.43). The median of the lowest net income class (9 people) was IDR 14.5 million with a total net income of IDR 43.50 million. The net income per person per day was IDR 13,170 (or US$0.99). The median of the highest net income class (12 people) was IDR 34.5 million with a total net income of IDR 138 million. The net income per person per day was IDR 31,525 (or US$ 2.37).

As Nurmanaf ^37^ observed, the levels of household income ranged from the first class, which was categorised as low income with a net household income of under US$1 per person per day, to the sixth class, which was categorised as high income with a net household income of more than US$1.7 per person per day. It can be concluded that 7% of fishers in Balikpapan Bay were categorised as having low level household income, 83% had a middle level household income, and 10% had a high level household income. In this study, it was calculated that the potential higher income that can be made from fishing, compared with harvesting wood, has resulted in the rate of overfishing reaching 37.3%.

**Figure 2.**
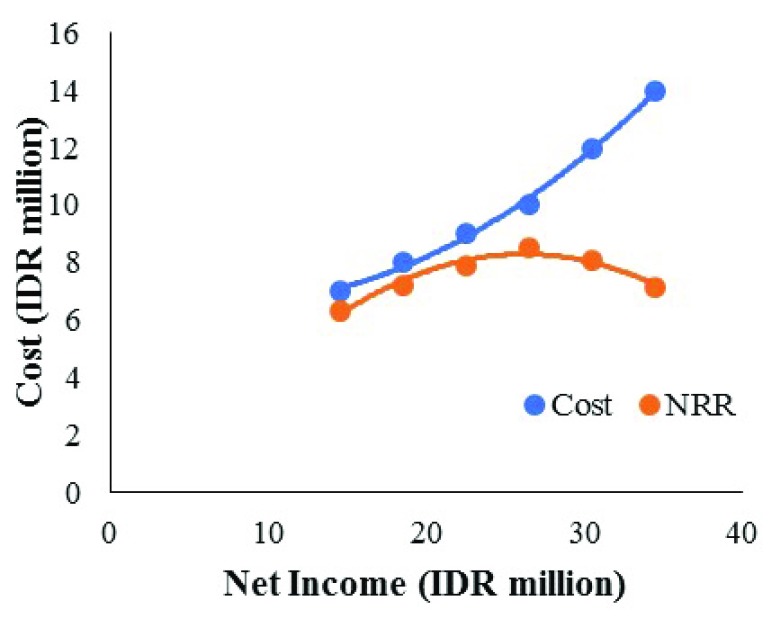
The curve of relationship between net benefit, cost of operation, and nominal rate of return (NRR) per month for fishers in Balikpapan Bay.

Table 2 and Figure 2 shows the relationship between net household income, cost of operation, and nominal rate of return (NRR) per month for fishers in Balikpapan Bay, as shown in table below. Table 2 and Figure 2 show that the total of cost operation that produce highest rate of NRR (11.4%) is IDR 10 million with a net benefit of IDR 26.5 million. This rate is high, compared to the short-term rate of return from the Indonesian Central Bank through the credit card bank rate of 3.5% per month.

**Table 2.** The phenomenon of diminishing marginal returns.

Net household income years ^-1^	Cost years ^-1^	NRR
14.5	7	9.8
18.5	8	10.5
22.5	9	11.0
26.5	10	11.4
30.5	12	11.1
34.5	14	10.9

Net household income years ^-1^ (million) IDR; cost years ^-1^, (million) IDR; NRR, nominal rate of return, %.

### Mangrove forests and ecosystem services

**Figure 3.**
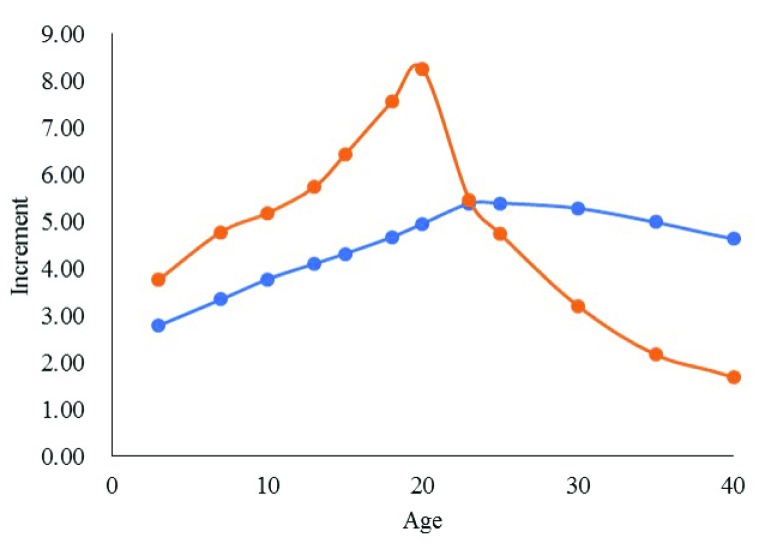
The curve of the relationship between growth increment and age.

Table 3 and Figure 3 shown the wood potential of mangrove forests for *Rhizophora apiculata* ranging in age from 3 to 35 years, as determined by the simulation described. Table 3 shows that the highest growth increment of wood production was reached at the age of 25 years, and the highest value of MAI was 5.39 m ^3 ^per hectare.

**Table 3.** The potential production of mangrove forests for *Rhizophora apiculata* wood (prior to thinning of trees). The number of mangrove trees decline gradually because of natural plant death.

Year	N	D	H	F	TV	MAI	PAI
3	2500	5.0	2.0	0.85	8.34	2.78	
7	1750	7.2	4.0	0.82	23.36	3.34	3.75
10	1700	8.4	5.0	0.8	37.66	3.77	4.77
13	1650	9.2	6.3	0.77	53.18	4.09	5.17
15	1600	9.9	7.0	0.75	64.63	4.31	5.72
18	1550	11.3	7.5	0.72	83.90	4.66	6.42
20	1390	12.0	9.0	0.7	98.99	4.95	7.55
23	1210	14.2	9.5	0.68	123.73	5.38	8.25
25	1100	15.0	10.5	0.66	134.64	5.39	5.46
30	950	17.5	11.0	0.63	158.27	5.28	4.73
35	940	18.5	11.5	0.60	174.26	4.98	3.20
40	870	19.4	12.0	0.60	185.07	4.63	2.16

Year, Age of trees; N, Population of Mangrove (trees ha ^-1^); D, Tree Diameter (cm); H, Branch-free Height (m); F, Trees Form Factor; TV, Total Volume (m ^3^ ha ^-1^); MAI, Mean Annual Increment (m ^3^ ha ^-1^ year ^-1^); CAI, Current Annual Increment (m ^3^ ha ^-1^ year ^-1^).

The restoration of mangrove forests in Balikpapan Bay produced a MAI for *Rhizophora apiculate* of 185.07 m ^3^ h ^-1^ for trees aged 40 years due to the high density of the mangrove trees. This result shows that the restoration of mangrove forests may produce a high value of ecosystem services for local communities. However, the optimal ecosystem service value will likely be achieved when the mangrove forests produces a MAI of 229.50 m ^3^ h ^-1^ for trees aged 40 years. Therefore, it is necessary to adopt tree thinning or tree clearing practices, thinning forest plots by 20% to 40% ( Table 4).

**Table 4.** The wood potential of mangrove forests for *Rhizophora apiculata* (after tree thinning).

Tree age, years	N	D	H	F	TV	MAI	PAI
18	1460	12.0	8.3	0.72	98.63	5.48	
20	1250	13.0	10.0	0.70	116.08	5.80	8.73
23	950	17.0	10.5	0.68	153.88	6.69	12.60
25	780	19.0	11.5	0.66	167.77	6.71	6.94
30	740	21.0	12.0	0.63	193.67	6.46	5.18
35	630	24.0	12.5	0.60	213.65	6.10	4.00
40	530	26.0	13.0	0.60	229.50	5.74	3.17

Year, Age of trees; N, population of mangrove (trees ha ^-1^); D, tree diameter (cm); H, branch-free height (m); F, trees form factor; TV, total volume (m ^3^ ha ^-1^); MAI, mean annual increment (m ^3^ ha ^-1^ year ^-1^); CAI, current annual increment (m ^3^ ha ^-1^ year ^-1^).

When measuring the benefits of ecosystem services, this study assumed that the net income per person income before tree thinning was less than US$1, while the net income per person after tree thinning was more than US$2. It is this low, pre-thinning income level for wood harvesting that has led to over-fishing, since fishing currently offers a higher income. Furthermore, the table shows that although the MAI for of 25-year-old trees was higher than that of trees aged 40 years (6.71 and 5.74, respectively), the total volume of 40-year-old trees was higher than that of 25-year-old trees (229.50 and 167.77, respectively).

## Conclusions

Since the mangrove is located in the primary trade and industry centers, there is a conflict of interest in land-used between huge companies and local communities that are economically dependent (e.g., fisherman). This study has provided some important conclusions regarding the restoration of mangrove forest in Balikpapan Bay. The study sampled 40 households (164 people) whose livelihood depends on the natural productivity of the mangrove forests in Balikpapan Bay, and additional 30 households (123 people) that were chosen via the accidental and snowball sampling method. The study showed that the total income from wood products is IDR 742,425,000 year ^-1^ or US $ 0.933 person ^-1^ day ^-1^ (300 ha × 98.99 × IDR 500,000, over 20 years). Furthermore, the total income from fishing is IDR 1,080,353,280 or US $ 1.43 person ^-1^ day ^-1^. Wood production provides a higher income to local community higher than fishing. Specifically, the income differences between wood production and fishing resulted in the rate of overfishing reaching 45.5%. The highest wood production was observed with 25-year-old trees, and the highest value of MAI was 5.39 m ^3 ^ha ^-1 ^for 40 years old trees. The results also suggest that tree thinning ranging, from 90 to 350 trees ha ^-1^, can increase the MAI value by around 24.5%.

## Data availability

Raw data associated with this study, including all de-identified questionnaires and raw data from thinning experiments, are available on OSF. DOI: https://doi.org/10.17605/OSF.IO/C6HFK ^36^.
