# Supplementary material for: Economic valuation from direct use of mangrove forest restoration in Balikpapan Bay, East Kalimantan, Indonesia
Source: F1000Res. 2019 Mar 29;8:9. Originally published 2019 Jan 3. [Version 2] doi: 10.12688/f1000research.17012.2 (PMC6456837; doi:10.12688/f1000research.17012.2)
Supplement: Peer review history

# Peer review: Reviewer response for version 2

**DOI:** 10.5256/f1000research.20432.r46487

**Published:** 2019-04-08

**Authors:** William L. Hargrove

**Affiliations:** 1 Center for Environmental Resource Management, University of Texas at El Paso, El Paso, TX, USA

Yes, I can change my recommendation to “Approved”.

I have read this submission. I believe that I have an appropriate level of expertise to confirm that it is of an acceptable scientific standard.

# Peer review: Reviewer response for version 2

**DOI:** 10.5256/f1000research.20432.r46486

**Published:** 2019-04-01

**Authors:** Roni Bawole

**Affiliations:** 1 Department of Marine Science, Faculty of Fisheries and Marine Science, University of Papua, Manokwari, Indonesia

My comments have been revised by the author. So this article can be indexed.

I have read this submission. I believe that I have an appropriate level of expertise to confirm that it is of an acceptable scientific standard.

# Peer review: Reviewer response for version 1

**DOI:** 10.5256/f1000research.18600.r43850

**Published:** 2019-02-06

**Authors:** Roni Bawole

**Affiliations:** 1 Department of Marine Science, Faculty of Fisheries and Marine Science, University of Papua, Manokwari, Indonesia

**Introduction:**

1. The concepts of ES and VE should be introduced briefly. Please explain what they mean and how they are defined related to mangrove forests in order to assess fishery activities.
2. What is the state of the knowledge of this research? From what I see I have found this research only reports what has been done by the project. So I suggest that the authors should be able to convey the novelty of this research. What's different about VE in Kalimantan from VE in Indonesia or elsewhere in the world?

 

**Materials and methods:**

 Please give more information about fishers, for example of their activities. Do they do one day fishing or not, and whether being fishermen is their main job, like many fishermen in Indonesia? This time is allocated for fishing for one week, month or year.

**Discussion:**

 The authors should also be able to discuss the relationship of mangroves ( *Rizophora apiculata*) with fisheries production based on location. Does low area abundance provide low fisheries production in areas with *Rizaphora*? This is seen from the EV approach.

**Conclusions:**

 Conclusions should be corrected or revised. I see that the current conclusion is still a summary of the methods and results and discussion. Summarize what has been learned and why it is interesting and useful.

I have read this submission. I believe that I have an appropriate level of expertise to confirm that it is of an acceptable scientific standard, however I have significant reservations, as outlined above.

# Response

**Published:** 2019-02-11

**Authors:** Bagus Nouval

Introduction:

1. The definition of ES has been described in the manuscript (refer to p.3), but we will add the definition of EV in the manuscript. Economic valuation (EV) is a quantitative assessment that is able to present the different range of values based on the specific method used, but it may allow fishermen to make decisions according to utilized goods and services provided by mangrove ecosystems.

2. The study is novel in that it examines the economic benefits of mangrove ecosystems. Since the mangrove is in the primary trade and industry centers, there is a conflict of interest in land-used between huge companies (e.g., mining, shipping, and cement) and local communities that are economically dependent (e.g., fishermen).

Materials and methods:

We already presented the criteria of fishermen activities in the manuscript at the section of data collection (refer to p. 4).

Discussion:

The relationship of mangrove trees ( *Rizophora apiculata*) and fishermen production is already discussed in the manuscript at the section of result and discussion (refer to p.5). The economic value produced by fishing is higher than harvesting wood of mangrove trees which is caused by overfishing.

Conclusions:

Since the mangrove is located in the primary trade and industry centers, there is a conflict of interest in land-used between huge companies (e.g., mining, shipping, and cement) and local communities that are economically dependent (e.g., fisherman). This issue becomes an interesting topic to be analyzed.

# Peer review: Reviewer response for version 1

**DOI:** 10.5256/f1000research.18600.r42539

**Published:** 2019-01-29

**Authors:** William L. Hargrove

**Affiliations:** 1 Center for Environmental Resource Management, University of Texas at El Paso, El Paso, TX, USA

This study focuses on mangrove forests, an important ecosystem in coastal areas. The study focuses on the economic value of wood production and also of fish production in brackish water ponds, which, along with overharvesting, contribute to the destruction of the mangrove ecosystem. Nonetheless some mangrove ecosystems have been restored through natural reseeding. The study provides interesting economic data on the value of mangrove forests. The impacts of thinning and also of restoring mangrove are assessed in economic terms, demonstrating that thinning can increase the economic value by about 25%. The economic value of fishing is also quantified. The greater economic value of fishing as measured in the daily income also contributes to overfishing. 

 My criticism of this paper is that the authors also discuss "ecosystem services", but seem to equate ecosystem services with the economic value of the wood or fish harvested. I consider ecosystem services to include other impacts related to water quality, habitat for flora and fauna, and other environmental impacts that are difficult to monetize. These are not really considered, only the economic value of wood production. Thus the authors should limit their discussion to the “economic services” of mangrove forests, and their contribution to the livelihoods of the local people. This, of course, does not diminish the importance of the mangrove forests as an economic resource, but evaluation of ecosystem services would require a different approach to evaluating all the environmental benefits of mangrove forests.

I have read this submission. I believe that I have an appropriate level of expertise to confirm that it is of an acceptable scientific standard, however I have significant reservations, as outlined above.

# Response

**Published:** 2019-02-05

**Authors:** Bagus Nouval

I agree with the suggestion from the referee. Since assessing ecosystem services provided ecological, sociocultural and economic human benefits, and monetary are essential, this research focused on examining the direct use of the natural products of the mangrove ecosystem in order to meet economic needs of the local community in Balikpapan bay, Indonesia. Identifying various mangrove ecosystem services, including wood production and fishing, which is consumed and sold at the local auction by local community leads this research to adopt economic value. Previous studies have adopted the economic value to evaluate ecosystem services. Therefore, the definition of ecosystem services in our manuscript will be limited on the economic services of mangrove ecosystem.
